# Pars Plana Vitrectomy and Evisceration Resulting in Death Due to Misdiagnosis of Retinoblastoma in Children: A Review of 3 Cases

**DOI:** 10.1097/MD.0000000000001338

**Published:** 2015-08-14

**Authors:** Tao Shen, Rongjiao Liu, Jing Lin, Huiqun Huang, Xiuling Li, Jianhua Yan

**Affiliations:** From the State Key Laboratory of Ophthalmology, Zhongshan Ophthalmic Center, Sun Yat-Sen University, Guangzhou, P. R. China.

## Abstract

Retinoblastoma is a curable intraocular malignancy in children. However, in clinical practice, retinoblastoma can sometimes be misdiagnosed and mismanaged, leading to extraocular extension and even death. In this report, a series of 3 cases are related that emphasize the conditions and consequences resulting from misdiagnosis and mismanagement of retinoblastoma. The clinical features, imaging findings, histopatholigical examination, and management in 3 case reports of children with misdiagnosed retinoblastoma are presented. Two of the cases received pars plana vitrectomy after being misdiagnosed with Coats disease or ocular blunt trauma, whereas the third case received evisceration after being misdiagnosed with suppurative endophthalmitis. When the diagnosis of retinoblastoma had been confirmed after a second surgery was performed in our hospital, only 2 of the cases received adjuvant orbital radiotherapy. All 3 cases died of systemic tumor metastases. Intraocular surgical procedures should be avoided in any equivocal case until the possibility of latent retinoblastoma is eliminated.

We strongly recommend that early enucleation be executed as soon as possible followed by postoperative adjuvant therapy under conditions wherein an intraocular surgery was inadvertently performed in an eye with retinoblastoma.

## INTRODUCTION

Retinoblastoma is the most common primary intraocular malignancy in children.^1,2^ The cure rate for this condition is as high as 96% in the USA because of early diagnosis and improved methods of treatment.^3^ However, a delay in diagnosis leads to poor survival rates as occurs in developing countries, especially in Asia and Africa, where mortality rates can reach 39% and 70%, respectively.^1–3^ In clinical practice, retinoblastoma can sometimes be misdiagnosed and mismanaged, leading to extraocular extension or even death. Here we present 3 cases of Chinese children referred to us for management of advanced retinoblastoma. Two of these cases were subjected to pars plana vitrectomy after being misdiagnosed with Coats disease or ocular blunt trauma. The third case was subjected to evisceration after being misdiagnosed with suppurative endophthalmitis. All 3 cases developed massive orbital extensions after intraocular surgeries and eventually died as a result of tumor metastases. Written informed consents were obtained from the participants or their guardians before the surgeries. This study was approved by the Institutional Review Board of the Zhongshan Ophthalmic Center, Guangzhou, China.

## CASE REPORTS

### Case 1

A 5-year-old girl presented with pain in the left eye (LE) accompanied by headache, nausea, and vomiting that were present for 11 months. The patient had been misdiagnosed with Coats disease and had received a pars plana vitrectomy combined with endophotocoagulation 1 year before admission to our hospital.

**FIGURE 1:**
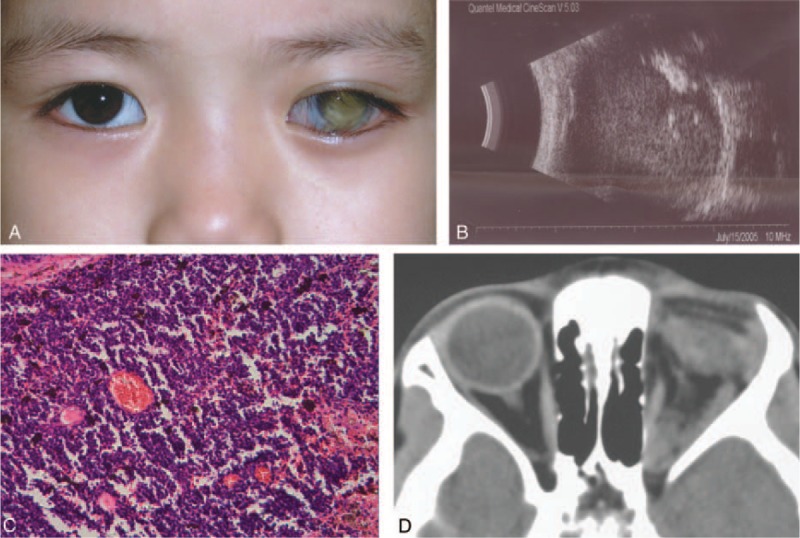
Clinical findings of case 1. (A) Clinical image shows pseudohypopyon in the left eye. (B) B-scan ultrasonography demonstrates a hyperechoic intraocular mass with calcification. (C) Histopathological examination (H&E stain, 100×) reveals poorly differentiated tumor cells, confirming the diagnosis of retinoblastoma. (D) Postoperative CT scan of the orbit shows a thickened left optic nerve (arrow). CT = computed tomography, H&E = hematoxylin and eosin.

On ocular examination, she had no light perception in the LE and 6/6 vision in the right eye (RE). There was secondary glaucoma present in the LE, with an intraocular pressure of T_+2_. Ectropion, mixed conjunctive congestion, band-shaped degeneration of the cornea, neovascularization of the iris, occlusion of the pupil, and opacification of the lens were also detected within the LE. The LE developed a pseudohypopyon resembling a masquerade syndrome (Figure 1A). Examination of the RE did not show any abnormalities in either the anterior or posterior segments. The B-scan ultrasonography of the LE showed a hyperechoic mass with calcification (Figure 1B).

Enucleation of the LE was performed during her hospitalization. Histopathological examination revealed a poorly differentiated retinoblastoma in the extraocular stage, showing combined endophytic and exophytic growth patterns, involving the choroid, sclera, optic disc, and optic nerve, and extended to the margins of resection (Figure 1C). Postoperative computed tomography (CT) scan of the orbit and brain showed thickening of the optic nerve (Figure 1D). A second-stage orbital exenteration or postoperative radiotherapy was suggested; however, the parents of the patient refused any further treatment. The patient expired after several months.

### Case 2

**FIGURE 2:**
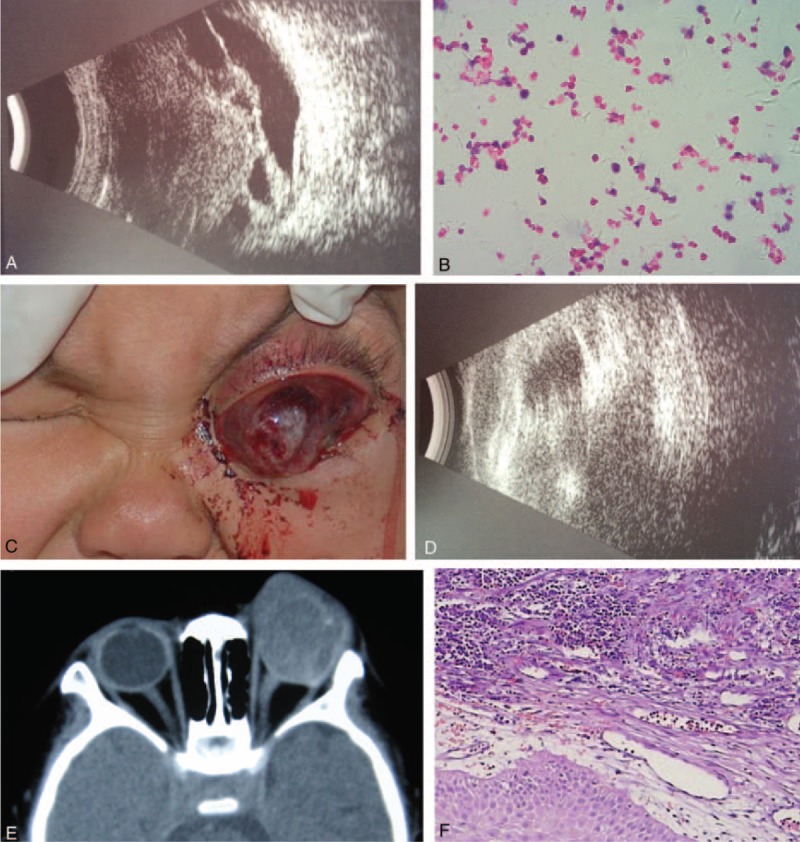
Clinical findings of case 2. (A) B-scan ultrasonography shows retinal detachment and vitreous opacity in the left eye. (B) Histopathological examination (H&E stain, 200×) of the fluid taken from intraocular surgery demonstrates tumor-like cells, suggesting an intraocular malignant tumor. (C) Clinical image of the patient reveals eyelid edema, severe subconjunctival hemorrhage, and opacification of cornea in the left eye, and a gray-white neoplasm with a size of 2.0 × 2.5 cm developing beside the left eye. (D) B-scan ultrasonography shows a hypoechoic lesion with a size of 2.4 × 2.2 cm, with no calcification detected. (E) CT scan reveals a left intraocular mass. (F) Histopathological examination (H&E stain, 100×) shows poorly differentiated tumor cells with local necrosis, confirming the diagnosis of retinoblastoma. CT = computed tomography, H&E = hematoxylin and eosin.

A 3-year-old girl was admitted presenting with LE pain for 2 months and proptosis for 1 week. The patient injured her LE in a fall 3 months earlier. She was diagnosed with ocular blunt trauma of the LE consisting of cataract, uveitis, retinal detachment, optic nerve contusion, and secondary glaucoma. The B-scan ultrasonography of the LE showed retinal detachment and vitreous opacity (Figure 2A). The patient was subjected to pars plana vitrectomy combined with par plana lensectomy, epiretinal membrane peeling, partial retinal incision, endophotocoagulation, and silicone oil tamponade. Histopathological examination of the intraocular tissue as assessed after vitrectomy suggested an intraocular tumor (Figure 2B), but no treatment was initiated.

Assessment of the patient during her stay at our hospital revealed that there was no light perception in the LE. The intraocular pressure of the LE was moderately elevated (T_+2_), whereas that of the RE was normal. On ocular slit lamp examination of the LE, eyelid edema, severe subconjunctival hemorrhage, and opacification of cornea were detected and a gray-white neoplasm with a size of 2.0 × 2.5 cm was observed beside the LE (Figure 2C). It was not possible to observe any of the remaining structures of the LE with any clarity. The anterior and posterior segments of the RE showed no abnormal findings. B-scan ultrasonography of the LE showed a hypoechoic lesion with a size of 2.4 × 2.2 cm, with no detectable calcification (Figure 2D). The CT scan also revealed the presence of a mass within the LE (Figure 2E).

Histopathological examination of the enucleated LE eyeball showed a poorly differentiated retinoblastoma with necrosis in the extraocular stage, which already involved the choroid, sclera, cornea, optic disc, and optic nerve, and extended to the margins of the resection (Figure 2F). The patient was administered a conventional dose of postoperative radiotherapy to the left orbit with a dose of 40 Gy 4 months later, followed by 6 courses of postoperative systemic chemotherapy. The patient expired at 16 months after pars plana vitrectomy because of metastasis of the retinoblastoma to the brain.

### Case 3

A 5-year-old boy was admitted presenting with a puffy eye and protrusion of ocular prosthesis in the LE that had been present for 2 months. The patient had been considered as having suppurative endophthalmitis when assessed at a local hospital 2 years previously. At that time, evisceration of ocular contents in the LE had been performed and combined with artificial eyeball implantation. No histopathological examination was executed.

**FIGURE 3:**
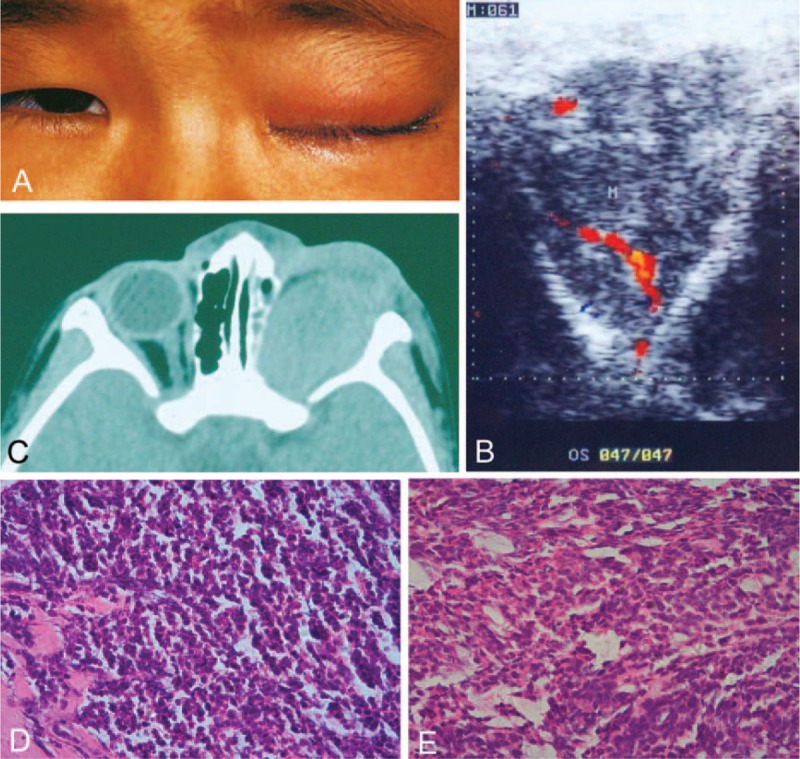
Clinical findings of case 3. (A) Clinical image shows swollen eyelids in the left eye. (B) Color Doppler ultrasonography shows a hypoechoic lesion with a size of 3.9 × 3.6 cm in the left orbit. (C) CT scan reveals an intraorbital mass of the left eye. (D) Histopathological examination (H&E stain, 200×) after subtotal resection indicates poorly differentiated neuroblastic cells, with minimal cytoplasm and basophilic nuclei. (E) Histopathological examination (H&E stain, 200×) after orbital exenteration in the left orbit confirms the diagnosis of retinoblastoma with poorly differentiated tumor cells and necrosis. CT = computed tomography, H&E = hematoxylin and eosin.

On ocular examination, no light perception was present in the LE because of the previous evisceration and accompanying eyelid swelling and edema of conjunctiva (Figure 3A). The RE had normal fixation without any abnormal changes. The color Doppler ultrasonography of the left orbit showed a hypoechoic lesion with a size of 3.9 × 3.6 cm (Figure 3B), and the CT scan revealed a left intraorbital mass (Figure 3C).

A subtotal resection of the intraorbital neoplasm was performed, followed by an orbital exenteration, because of the histopathological report indicating a malignancy as revealed after the subtotal resection (Figure 3D). Histopathological examination confirmed the diagnosis of retinoblastoma with poorly differentiated tumor cells and necrosis involving the periphery of the mass (Figure 3E). Radiotherapy was administered to the left orbit with a dose of 50 Gy 2 months later. The patient expired almost 2 years later due to metastasis of malignancy to the brain. The parents related that the metastatic tumor also involved the left foot of the patient.

## DISCUSSION

Retinoblastoma is typically a disease of early childhood, with most patients being recognized by 3 years of age.^4^ Generally, these patients present with leukocoria in 60% and with strabismus in 20% of the cases.^5^ The typical ophthalmoscopic appearance of the tumor is that of a unifocal or multifocal whitish retinal mass with rich irregular vessels.^2^ Retinoblastoma may be endophytic, with tumor growth into the vitreous in 60%, or exophytic, with growth into the subretinal space in 39% of the cases.^4,6^ Atypical manifestations of retinoblastoma are similar to other diseases such as Coats disease, endogenous endophthalmitis, retinopathy of prematurity, familial exudative vitreoretinopathy, and orbital cellulitis. As a result, these children may be misdiagnosed leading to various kinds of intraocular surgeries, including cataract surgery, par plana lensectomy, par plana vitrectomy, or evisceration of ocular contents. Such procedures may result in a delay in establishing the correct diagnosis and, of greater concern, predispose these patients to extraocular extension of the retinoblastoma.^7^

Coats disease characteristically causes a chronic, unilateral, progressive retinal detachment associated with massive subretinal exudation from permeable telangiectatic retinal blood vessels.^4^ This condition commonly manifests at 6 to 8 years of age and is more common in men, whereas retinoblastoma often manifests prior to age 5 years and shows no sex difference. However, it is sometimes difficult to differentiate retinoblastoma from Coats disease based on both clinical features and image findings. Both conditions can show intraocular masses, associated with limited calcification and secondary retinal detachment. Case 1 of this study was misdiagnosed as Coats disease and received vitrectomy. An additional unfortunate situation can involve a child with an eye injury, as in case 2. Such a patient would usually be diagnosed as having traumatic hyphema, vitreous hemorrhage, or retinal detachment, and the more lethal condition of retinoblastoma may be overlooked. As a result, the par plana vitrectomy can lead to extraocular extension. Shields et al^8^ analyzed 11 patients who had undergone vitrectomy on an eye with unsuspected retinoblastoma. The main preoperative diagnoses included vitreous hemorrhage in 7 patients (64%), toxocariasis in 2 patients (18%), toxoplasmosis in 1 patient (9%), and endophthalmitis in 1 patient (9%). Enucleation was necessary in all 11 patients, combined with adjuvant treatment of radiotherapy and chemotherapy. Only 1 patient died of metastatic retinoblastoma 24 months later.

Vitrectomy or pars plana vitrectomy represent aggressive modalities for mechanical removal of intraocular retinoblastoma because of the risk of tumor cell dispersion from the sclerotomies and recurrence of the intraocular tumor.^9^ Accordingly, it is not usually applied in the treatment of retinoblastoma. However, when the intraocular tumor has been controlled or successfully managed, vitrectomy can provide a means for managing complications of retinoblastoma, including retinal detachment and vitreous hemorrhage. Vitrectomy is also applicable for localized vitreous seeds of retinoblastoma, although it should be used judiciously because of the risk of tumor cell dispersion. Moreover, use of vitrectomy requires that the retinal tumor has been stable for a sufficient period of time before vitrectomy and a limited number of vitreous seeds are present.^9,10^ The potential for orbital recurrence of retinoblastoma is unlikely, unless intraocular surgeries (eg, pars plana vitrectomy) were performed prior to enucleation without a diagnosis of retinoblastoma.^11,12^

Endophthalmitis, which can produce a “masquerade syndrome,” is sometimes confused with retinoblastoma because of leukocoria, hypopyon, or hyphema, as in our case 3. Infectious causes of endophthalmitis in patients <3 years of age include bacteria, congenital toxoplasmosis, toxocariasis, syphilis, and herpes simplex virus. Typically, in children with endophthalmitis, a previous ocular injection, pain, hypopyon, and elevated intraocular pressure are the main clinical findings. Such symptoms are all quite different from those of retinoblastoma. In our case 3, the ocular contents were eviscerated, rather than enucleation, which exacerbated tumor extraocular extension and resulted in massive orbital infiltration. Moreover, the lack of any histopathological examination of the ocular contents contributed to a 2-year delay in the diagnosis of retinoblastoma. Therefore, ocular content evisceration is prohibited in the treatment of retinoblastoma because of the high risk of tumor extension. In any case of unilateral leukocoria with signs of intraocular inflammation, the diagnosis of retinoblastoma must be considered.^7^ If an intraocular surgical procedure has been performed in an equivocal case, histopathological analysis of the intraocular tissue is strongly suggested, and enucleation should be performed as soon as possible after a diagnosis of retinoblastoma has been established.^11,12^

In this report, 3 cases of orbital metastasis of retinoblastoma were presented to our department. In all cases, an intraocular surgery had been performed prior to establishing a diagnosis of retinoblastoma and no specific treatment for reducing the subsequent recurrence likelihood of retinoblastoma was initiated. When the diagnosis of retinoblastoma had been confirmed after the second surgery in our hospital, the patient of case 1 refused any adjuvant treatments. Although cases 2 and 3 did have adjuvant orbital radiotherapy at their local hospitals, these postoperative radiotherapies were delayed for 2 and 4 months, respectively, and only case 2 received systemic chemotherapy to prevent tumor dissemination. Sadly, all 3 children died of systemic tumor metastases.

In summary, retinoblastoma may present with atypical features such as vitreous hemorrhage or signs of vitreous inflammation. Vitrectomy should be avoided in these cases until the possibility of latent retinoblastoma has been eliminated.^8^ If an intraocular surgical procedure is performed in an eye with suspected retinoblastoma, enucleation combined with adjuvant chemotherapy or orbital radiotherapy should be performed in the early postoperative period.^8,11^ We strongly recommend postoperative adjuvant therapy, even when there is no histopathological evidence of local extraocular tumor spread.^11^
